# Cardiosphere-Derived Cells Improve Function in the Infarcted Rat Heart for at Least 16 Weeks – an MRI Study

**DOI:** 10.1371/journal.pone.0025669

**Published:** 2011-10-17

**Authors:** Carolyn A. Carr, Daniel J. Stuckey, Jun Jie Tan, Suat Cheng Tan, Renata S. M. Gomes, Patrizia Camelliti, Elisa Messina, Alessandro Giacomello, Georgina M. Ellison, Kieran Clarke

**Affiliations:** 1 Department of Physiology, Anatomy and Genetics, University of Oxford, Oxford, United Kingdom; 2 National Heart and Lung Institute, Imperial College, London, United Kingdom; 3 Cluster of Cardiovascular Science, Advanced Medical and Dental Institute, Universiti Sains Malaysia, Penang, Malaysia; 4 Department of Experimental Medicine, Cenci-Bolognetti Foundation, Pasteur Institute, University La Sapienza, Rome, Italy; 5 Stem Cell and Molecular Physiology Laboratory, Liverpool JM University, Liverpool, United Kingdom; Centro Cardiologico Monzino, Italy

## Abstract

**Aims:**

Endogenous cardiac progenitor cells, expanded from explants via cardiosphere formation, present a promising cell source to prevent heart failure following myocardial infarction. Here we used cine-magnetic resonance imaging (MRI) to track administered cardiosphere-derived cells (CDCs) and to measure changes in cardiac function over four months in the infarcted rat heart.

**Methods and Results:**

CDCs, cultured from neonatal rat heart, comprised a heterogeneous population including cells expressing the mesenchymal markers CD90 and CD105, the stem cell marker c-kit and the pluripotency markers Sox2, Oct3/4 and Klf-4. CDCs (2×10^6^) expressing green fluorescent protein (GFP+) were labelled with fluorescent micron-sized particles of iron oxide (MPIO). Labelled cells were administered to the infarcted rat hearts (n = 7) by intramyocardial injection immediately following reperfusion, then by systemic infusion (4×10^6^) 2 days later. A control group (n = 7) was administered cell medium. MR hypointensities caused by the MPIOs were detected at all times and GFP+ cells containing MPIO particles were identified in tissue slices at 16 weeks. At two days after infarction, cardiac function was similar between groups. By 6 weeks, ejection fractions in control hearts had significantly decreased (47±2%), but this was not evident in CDC-treated hearts (56±3%). The significantly higher ejection fractions in the CDC-treated group were maintained for a further 10 weeks. In addition, CDC-treated rat hearts had significantly increased capillary density in the peri-infarct region and lower infarct sizes. MPIO-labelled cells also expressed cardiac troponin I, von Willebrand factor and smooth muscle actin, suggesting their differentiation along the cardiomyocyte lineage and the formation of new blood vessels.

**Conclusions:**

CDCs were retained in the infarcted rat heart for 16 weeks and improved cardiac function.

## Introduction

The optimum stem cell for treatment of the infarcted heart has yet to be established. Despite promising studies using bone marrow cells in animal models, the results from the clinical trials have not been conclusive [1], causing a shift in interest to endogenous cardiac stem cells, which were first identified in 2003 [2], [3]. Isolation and culture of these cells from biopsy samples, *via* the production of cardiospheres, was reported by Messina *et al* in 2004 [4]. Further expansion of cardiosphere-derived cells (CDCs) as an adherent monolayer [5] generated a mixed population, comprising c-kit+ cardiac progenitor cells and CD90+ cardiac mesenchymal cells, and provided a sufficient number of cells for therapy within 1–2 months. Promising data from animal studies [5], [6] lead to a clinical trial (CADUCEUS; see ClinicalTrials.gov for details). However, the characteristics and efficacy of explant-derived cells (EDCs) has been questioned [7], [8], [9], with the suggestion that outgrowth cells from explants were haematological cells [7], [9] and that EDCs were not retained in the heart following administration [8]. In response, the clonogenicity, multipotency and capacity for self-renewal of EDCs and CDCs were demonstrated [10], [11], [12]. Furthermore, improved ejection fraction has been reported in the infarcted rat heart at 6 weeks after administration of CDCs or EDCs [12]. As with other stem cell types [13], the observed improvement in cardiac function with stem cell therapy utilising cardiac-derived cells has been ascribed both to differentiation of donor cells, providing new myocytes and blood vessels, and to the release of paracrine factors, improving cardiomyocyte survival, activating endogenous cardiac stem cells and angiogenesis [11].

Here CDCs cultured from neonatal rat heart, and characterised using immunocytochemistry, flow cytometry and quantitative RT-PCR, were labelled with fluorescent MPIOs and with DiI cell tracker dye and administered to the infarcted rat heart following reperfusion. The limited retention of administered cells has beset stem cell studies for the heart, irrespective of the cell type used. Furthermore, the best mode and timings of injection have yet to be established. Therefore CDCs were administered twice, by direct injection at the time of coronary artery ligation and by systemic infusion after 2 days, thereby maximising the potential of the CDCs to improve function in the infarcted heart. High resolution MRI was used to track MPIO-labelled cells and to measure *in vivo* cardiac function at baseline and at regular intervals over a long-term follow up of 4 months following cell administration. Use of a cardiac progenitor cell, a model of infarction/reperfusion and high resolution non-invasive MRI, has allowed us to further validate the potential of CDCs by showing that CDCs are retained in the heart for at least 16 weeks and we show for the first time that CDC therapy provides long-term improvement in cardiac function.

## Materials and Methods

### Animals

Sprague Dawley rats (Harlan, UK) and Sprague Dawley SD-Tg(GFP)2BalRrrc rats (SD-GFP; Rat Resource and Research Centre, Missouri) were allowed free access to standard rodent chow and water throughout the study. All investigations conformed to the Guide for the Care and Use of Laboratory Animals published by the US National Institutes of Health (NIH Publication No. 85-23, revised 1996), the Home Office Guidance on the Operation of the Animals (Scientific Procedures) Act, 1986 (HMSO), and to institutional guidelines. Approval was granted by the University of Oxford Animal Ethics Review Committees and the Home Office (Project Licence numbers 30/2278 and 30/2755).

### Rat CDC isolation and culture

Rat CDCs were cultured according to the method of Smith *et al* [5]. SD-GFP neonatal rats were sacrificed by cervical dislocation. Hearts were isolated, washed with Dulbecco's phosphate buffered saline (DPBS; Invitrogen) and minced into small explant pieces in 0.05% trypsin-EDTA (Invitrogen). Explants were plated on fibronectin-coated petri dishes with 1.5 ml of complete explant medium (CEM) comprising Iscove's modified Dulbecco's medium (IMDM; Invitrogen) supplemented with 20% foetal bovine serum (FBS; Invitrogen), 1 U/ml penicillin, 1 ug/ml streptomycin and 0.2 mM L-glutamine (Gibco). After 3–4 days in 5% CO_2_ at 37°C, small, round, phase bright cells grew out from the explants over a bed of stromal-like cells. Once they reached 80–90% confluency, these explant-derived cells (EDCs) were isolated using trypsin and re-plated on poly-d-lysine coated 24 well plates in cardiosphere growth medium (CGM) comprising 65% Dulbecco's modified eagle medium (DMEM/F12), 35% IMDM, 7% FBS, 2% B27 (Invitrogen), 25 ng/ml cardiotrophin (Peprotech EC), 10 ng/ml epidermal growth factor (EGF; Peprotech EC), 20 ng/ml basic fibroblast growth factor (FGF; Promega) and 5 units thrombin (Sigma). EDCs could be harvested every 7 days, for up to 4 weeks.

Cardiospheres formed after 2–3 days, when they were harvested by mechanical trituration and plated in CEM in fibronectin-coated flasks. Cardiosphere-derived cells (CDCs) were passaged every 5–7 days to passage 2. Secondary cardiospheres could be formed by replating CDCs in CGM in poly-lysine coated 24 well plates.

### CDC labelling

CDCs were incubated overnight with micron-sized particles of iron oxide (MPIO; 2 µl/cm^2^, encapsulated magnetic microspheres, Bangs Laboratories Inc.), on the day prior to transplantation. Viability and proliferation of MPIO-labelled CDCs was measured over 3 days using the LIVE/DEAD cell cytotoxicity kit (Molecular Probes). For the *in vivo* study, MPIO-labelled CDCs were washed twice with PBS and stained with DiI (Molecular Probes) in PBS for 30 minutes before trypsinization. Approximately 2×10^6^ cells were suspended in 50 µl CEM for direct injection to the myocardium and 4×10^6^ cells were suspended in 500 µl CEM for systemic infusion. For MR microscopy, MPIO-labelled CDCs were suspended in 1% agarose doped with gadolinium DTPA (4 µl/ml).

### CDC differentiation into cardiomyocytes

CDCs were plated in 6 well plates coated with 0.1% gelatin at a density of 10,000 cells/cm^2^ and incubated overnight in CEM. Medium was changed to differentiation medium (45% IMDM, 45% DMEM, 10% ESQ FBS (Invitrogen), Insulin-transferrin-selenium (1×; Invitrogen) and 300 µM ascorbic acid) for 6–8 hours, followed by incubation with differentiation medium containing 1 µM dimethyl sulphoxide (DMSO) for 10 days, with fresh medium added every 2 days.

### Characterisation of CDCs using qRT-PCR

Primers were designed using Primer3 software based on interpretation of GenBank or Ensembl Genome Browser search results and are listed in File S1. Total RNA was extracted using Trizol (Sigma) and treated with Turbo DNA-free (Ambion). Complementary DNA (cDNA) was synthesized from the treated RNA template using AB high capacity transcriptase kit (Applied Biosystem). Real time PCR amplification was performed (AB StepOnePlus Real-Time, CA) and all data were analyzed using the published 2^−ΔΔCt^ method [14] with the StepOne modification where the amplification efficiencies of the target and reference genes were not equal. Relative mRNA levels were normalized to GAPDH and expressed relative to neonatal whole heart total RNA. Further details are provided in File S1.

### Characterisation of CDCs using immunocytochemistry

Cells (8×10^4^/80 µl) cultured on chamber slides were fixed with 4% PFA on ice for 20 minutes. For intracellular staining, cells were permeabilized with 0.1% triton X in PBS for 10 minutes. This step was omitted for cytoplasmic staining. Then cells were blocked with 10% donkey serum for 30 minutes and stained with primary antibody (see File S1) overnight at 4°C. After rinsing, cells were labelled with secondary antibody for one hour at 37°C, counterstained with 4′,6-diamidino-2-phenylindole (DAPI, Sigma) and mounted with VECTASHIELD® mounting medium. Negative control staining was performed whereby the primary antibody was omitted. Immunostaining was visualized and analyzed using laser scanning confocal microscopy (Zeiss LSM510 and LSM710 Meta laser confocal microscope). For quantification of cardiac troponin I (cTnI) staining, the percentage of positively stained cells was counted in 18 representative fields from 3 experiments (at 400× magnification).

### Rat myocardial infarction and CDC administration

The left anterior descending (LAD) coronary artery of female SD rats (200–250 g, n = 14) was occluded using the method of Michael *et al* [15]. In brief, following anaesthesia, using 2% isoflurane in O_2_, and thoracotamy, the pericardium was removed and a 5-0 prolene suture placed under the LAD, about 2 mm from the origin. The suture was tied around a small piece of PE tubing, occluding the LAD, and the chest closed. After 50 minutes, the chest was re-opened and the tubing removed to allow reperfusion. Ten minutes after ischaemia/reperfusion, CDCs (2×10^6^ in 50 µl CEM; n = 7; CDC-treated group) or medium (50 µl CEM; n = 7; Control group) were injected over four sites in the peri-infarct region. Two days after MI, a bolus of CDCs (4×10^6^ in 500 µl CEM) or medium (500 µl CEM) was administered *via* the tail vein to the CDC-treated and control group, respectively. In sham animals (n = 3), the thoracotamy was performed but no stitch placed in the heart.

### Cardiac cine MRI

Cardiac cine MRI was performed as previously described [16]. Briefly, rats were anaesthetised with 2.5% isoflurane in O_2_, positioned supine in a purpose built cradle and lowered into a 60 mm birdcage coil in a vertical bore 500 MHz, 11.7 T MR system with a Bruker console running Paravision 2.1.1. A stack of 7–8 contiguous 1.5 mm true short axis ECG-gated cine images were acquired to cover the entire left ventricle. The epicardial and endocardial borders were outlined in end diastolic and systolic frames using the freehand drawing tool in Image J [17]. Cardiac mass and left ventricular volumes were summed over the whole heart. Further details are provided in File S1. Animals were sacrificed by overdose of pentobarbitone after the final MR images were acquired.

### High resolution 3D MR microscopy

Hearts (n = 4; 2 hearts from each group) were isolated and fixed in 4% (w/v) paraformaldehyde (Sigma, UK) in phosphate buffered saline (pH 7.2) and embedded in 1% (w/v) agarose doped with gadolinium DTPA in a 20 mm NMR-tube. High-resolution MRI was performed in a 20 mm quadrature-driven birdcage coil (Rapid Biomedical, Würzburg, Germany) using a fast gradient echo sequence (see File S1for details).

### Histological analysis

Infarcted hearts (n = 10; 5 hearts from each group) were isolated, cut into basal and apical halves and frozen in OCT (Tissue-Tek) over dry ice. Frozen sections were cut into serial 10 µm slices and stained with or picro-Sirius Red. The amount of positive staining was quantified using ImagePro Plus 5.0 (MediaCybernetics Inc, Bethesda, Md) image analysis software. Infarct volume was calculated by multiplying the area of infarcted tissue in each tissue slice by the distance between those slices in the heart (1.5 mm).

### Immunohistochemistry

#### Capillary density

In a humidity chamber, tissue sections were fixed in 4% paraformaldehyde for 10 minutes at room temperature, washed and permeabilized with 0.5% Triton for 10 minutes at room temperature. Sections were incubated for 20 minutes with diluted normal mouse blocking serum and then with PECAM-1/CD31 mouse anti-rat (AbD Serotec, MCA1334G; 1∶100 dilution), for 1 hour at room temperature. The sections were washed and incubated with biotinylated anti mouse secondary antibody (Vectastain ABC kit; Vector Laboratories, PK-4002) for 30 minutes at room temperature, followed by incubation with DAB Perosidase Substrate kit (Vector Laboratories, SK-4100) for 10 minutes and mounting. Images were acquired on ×10 magnification with a GETI light microscope connected to Cannon Digital camera. CD31 was quantified using ImageJ.

#### Cell characterization

Frozen tissue sections were fixed using ethanol-glycine fixative for 20 minutes at room temperature and blocked with 10% donkey serum in PBS for 30 minutes. Tissue sections were incubated with primary antibody (see File S1) at 4°C overnight. After rinsing, tissue was labelled with secondary antibody for 1 hour, counterstained with DAPI and mounted with VECTASHIELD® mounting medium. Negative control staining was performed whereby the primary antibody was omitted. Immunostaining was visualized and analyzed using laser scanning confocal microscopy (Zeiss LSM510 and LSM710 Meta laser confocal microscope). The proportion of cells labelled with MPIO that also stained for cTnI, SMA or vWF was quantified in the infarct/peri-infarct region of the heart from at least 10 representative fields from 3 hearts (at 63× magnification). A capillary was defined as 1 or 2 endothelial cells spanning the vWF-positive vessel circumference. An arteriole was defined as 2 or more SMA positive cells spanning the vessel circumference.

### Statistical analysis

Results are presented as means ± standard errors. Differences were considered significant at p<0.05, determined using analysis of variance with a post hoc *t*-test with Tukey correction or a Student's *t*-test (SPSS).

## Results

### CDC culture and characterisation

**Figure 1:**
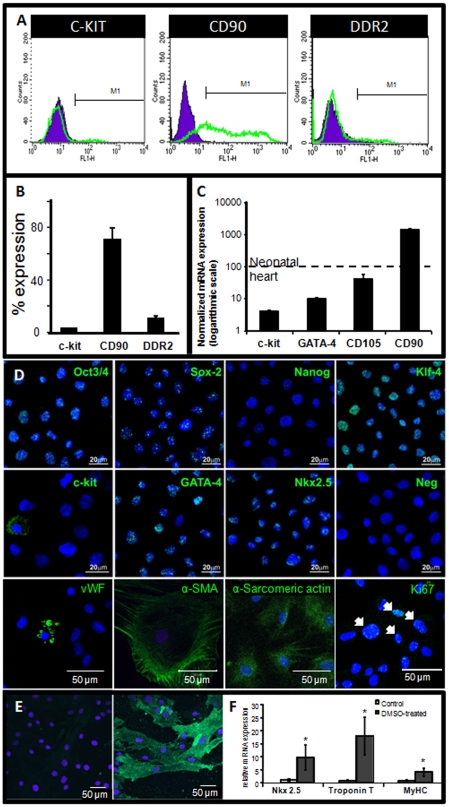
Characterisation of CDCs. Surface marker expression and mRNA in passage 2 CDCs was measured using (A, B) flow cytometry for c-kit, CD90 and DDR2, representative plots are shown in (A) and quantication in (B); (C) qRT-PCR for c-kit, GATA-4, CD105 and CD90, expressed relative to 100% expression in neonatal heart and normalised to GAPDH; and (D) immunocytochemistry for Oct3/4, Sox2, Nanog, KLF-4, c-kit, GATA-4, Nkx2.5, von Willibrand factor (vWF), α–smooth muscle actin (α–SMA), α–sarcomeric actin and Ki67. (E & F) Incubation with DMSO for 10 days promoted differentiation along the cardiac lineage as shown by increased protein expression of cTroponin I (E: green) and mRNA expression of Nkx2.5, Troponin T and myosin heavy chain (MyHC) (F).

Explant-derived cells (EDCs) were harvested and cultured to form cardiospheres, which were then expanded as a monolayer culture to passage 2 (p2) as CDCs within 3–4 weeks of biopsy. Flow cytometry revealed that 3.0±0.1% of p2 CDCs expressed the stem cell marker c-kit, 71±7% expressed CD90 and 10±2% expressed the fibroblast marker, DDR2 (Figure 1A & 1B), indicating a largely mesenchymal cell population (figure S1). Less than 1% of CDCs expressed CD45 (data not shown). Gene expression was analysed using quantitative RT-PCR and expressed relative to expression in neonatal rat heart (Figure 1C). CDCs expressed c-kit, the cardiac transcription factor GATA-4 and the glycoprotein CD105 (4.1±0.3%, 10.2±0.7% and 42±17% of that in neonatal heart, respectively) with high levels of CD90 (14±1 fold higher than in neonatal heart.

Using immunocytochemistry, cells were found to express the pluripotency markers, Oct3/4, Sox2, and Klf-4, with little or no expression of Nanog (Figure 1D). In line with the flow cytometry data, immunocytochemistry showed that approximately 2% of CDCs expressed c-kit (Figure 1D). Cardiosphere-derived cells expressed the cardiac transcription factors, GATA-4 and Nkx2.5 indicative of their origin and also showed multipotency through spontaneous differentiation into the three main cardiac lineages; endothelial (positive for von Willibrand factor (vWF)), smooth muscle (positive for α-smooth muscle actin (SMA)) and cardiomyocyte (positive for α-sarcomeric actin) (Figure 1D). Cycling cells were detected by staining for Ki67 (Figure 1D). CDCs cultured to full confluency showed increased expression of α-sarcomeric actin and began to express cardiac troponin T (figure S2). Culture in differentiation medium containing DMSO for 10 days promoted CDC differentiation further along the cardiomyocyte lineage, detected by immunostaining for cardiac troponin T and increased mRNA expression of Nkx2.5, cardiac troponin T and myosin heavy chain. (Figure 1E and Figure S2). Cells treated with DMSO began to adopt an elongated morphology more indicative of a cardiomyocyte and to show striations not evident in the cells cultured beyond confluency (figure S2).

Although the CD90+ population may include cardiac fibroblasts [7], only 10% of CDCs expressed the fibroblast marker DDR2 [18] and the detection of Oct 3/4, Sox2 and Klf-4 confirmed the presence of pluripotent stem cells within the CDC population. Furthermore, treatment with DMSO induced differentiation and expression of cTnT in a higher percentage of cells (19±6%) than the proportion of c-kit+ cells present (3.0±0.1%) suggesting that the CDC population contains a c-kit negative population of cells capable of differentiation into cardiomyocytes.

### MPIO labelling

**Figure 2:**
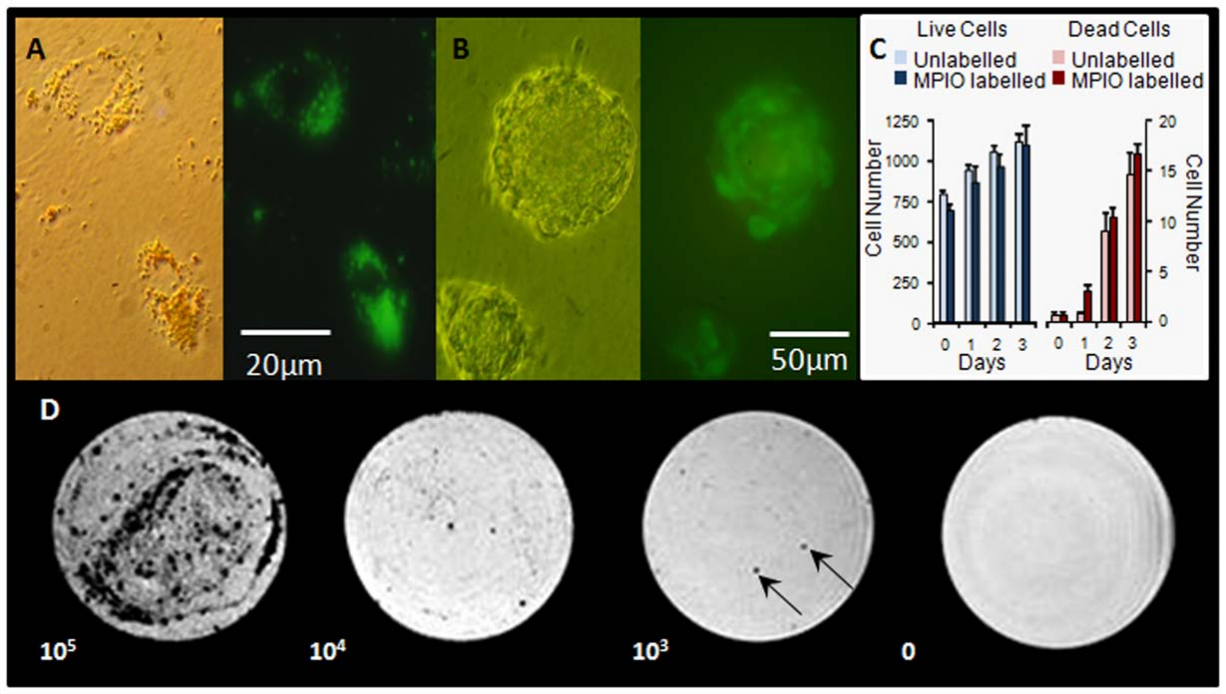
CDCs were not adversely affected by labelling with MPIOs and could be detected using MR microscopy. (A) CDCs endocytosed fluorescent MPIOs during overnight incubation; (B) MPIO-labelled CDCs retained the ability to form cardiospheres; (C) live (blue) and dead (red) cell numbers were comparable between unlabelled and MPIO-labelled CDCs; (D) MPIO-labelled CDCs could be detected at the single cell level (10^3^ cells / 0.5 µl, arrows) using MR microscopy.

The viability of the CDCs was not altered after labelling with fluorescent microparticles of iron oxide (MPIO; Figure 2A). MPIO-labelled cells formed secondary cardiospheres (Figure 2B) and proliferated at a rate comparable with unlabelled CDCs (Figure 2C). MPIO-labelled CDCs were imaged using MR with a 4×10^6^ voxels/ml resolution. It was found that 10^5^ labelled cells, suspended in 0.6 ml agarose, caused a large signal void (Figure 2D). When only 1% of the 10^5^ CDCs were MPIO-labelled, small regions of signal void were detected throughout the phantom, indicating that the labelled cells could be detected with single cell resolution.

### Cardiac function

**Figure 3:**
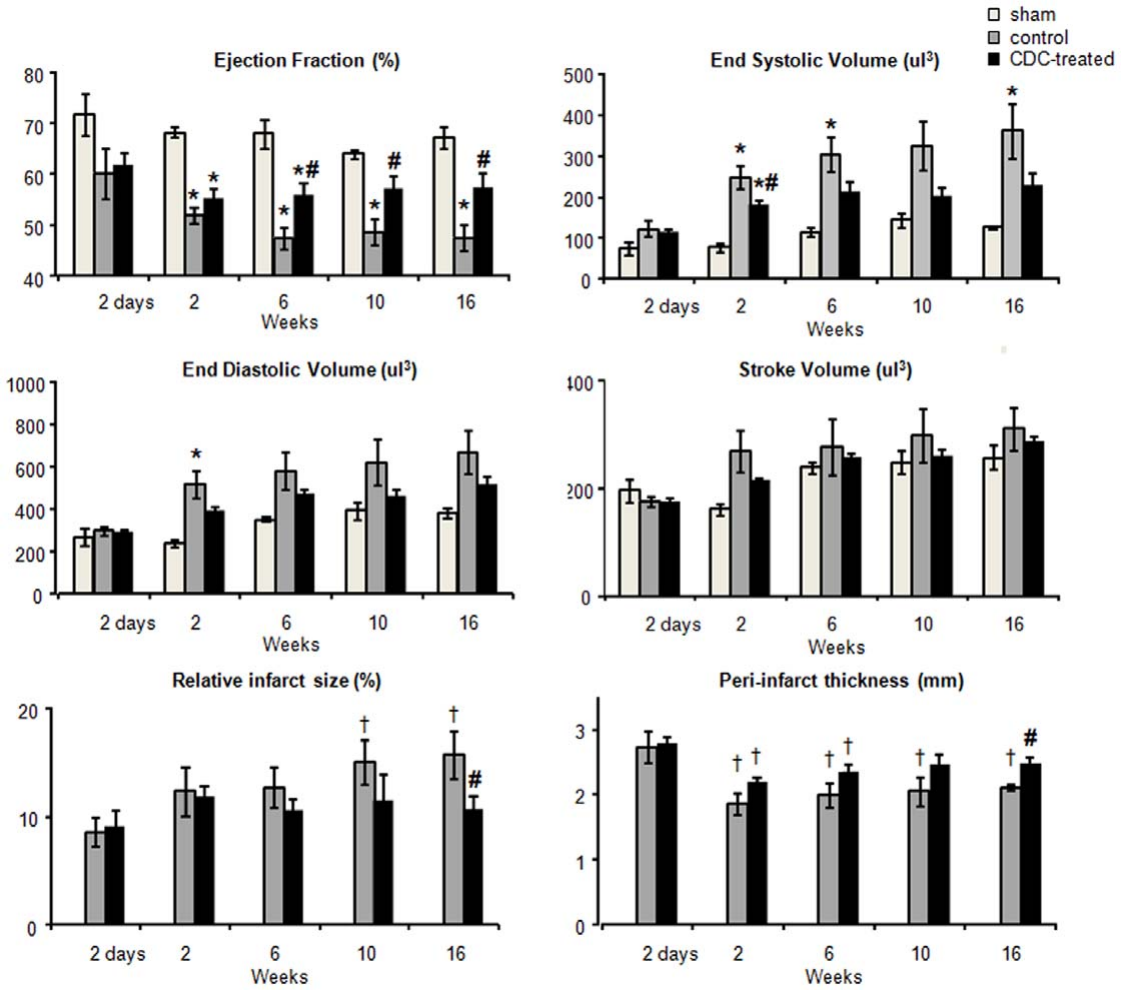
CDC administration improved cardiac function. Cardiac ejection fraction, end systolic volume, end diastolic volume, stroke volume, relative infarct size and end-systolic peri-infarct thickness, measured over 16 weeks using MRI, showed that cardiac function in the infarcted hearts was improved by administration of CDCs. * p<0.05 vs sham ; # p<0.05 vs control; † p<0.05 vs 2 days (shown only for infarct size and peri-infarct thickness for clarity).

Cine-MRI was used to measure cardiac morphology and function at baseline (2 days post MI) and at 2, 6, 10 and 16 weeks after MI and the administration of CDCs (Figure 3). At baseline, there were no significant differences between control and CDC-treated hearts in ejection fraction (EF), end diastolic volume (EDV), end systolic volume (ESV), stroke volume (SV) or cardiac output (CO) (see Figure 3 and table S1). In control hearts, cardiac remodelling resulted in a significant decrease in EF between baseline and 2 weeks and again between 2 and 6 weeks, such that, by 6 weeks, control heart EF had decreased by 13% (EF 47±2%, p<0.05 vs. baseline) and was significantly lower than that of CDC-treated hearts (EF 56±3%, p<0.05 vs. control). This 9% difference in ejection fraction was maintained to 16 weeks post MI such that the ejection fractions of CDC-treated hearts were not significantly different from shams (EF sham 67±2%; control 47±3% p<0.05 vs sham; CDC 57±3%, p<0.05 vs control, ns vs sham; Figure 3). Although both control and CDC-treated hearts showed significant dilation by two weeks, the end systolic volume of the CDC-treated hearts was significantly smaller at 2 weeks than that of the control hearts (ESV sham 76±9 mm^3^, control 247±27 mm^3^, p<0.05 vs sham; CDC 177±13 mm^3^, p<0.05 vs sham and control). Further remodelling was attenuated in CDC-treated hearts, compared with control hearts, such that at later time points end systolic volumes of control hearts, but not of CDC-treated hearts, were significantly larger than those of shams (Figure 3). Relative infarct size (measured as the area of the myocardial wall that was akinetic) was the same in all infarcted hearts at 2 days (control 24±4 mm^2^, 9±1% of LV wall, CDC-treated 24±4 mm^2^, 9±2%), and by 10 weeks had increased significantly in control hearts but not in CDC-treated hearts (control 56±4 mm^2^, 15±2%, p<0.05 vs baseline; CDC-treated 38±4 mm^2^, 12±2%, ns vs baseline; Figure 3). At 16 weeks, the infarct size in CDC-treated hearts was significantly lower than that in control infarcted hearts (control 61±9 mm^2^, 16±2%, CDC-treated 36±7 mm^2^, 10±1%, p<0.05 vs control; Figure 3). End systolic posterior wall thickness was comparable between the three groups. At 2 and 6 weeks, the peri-infarct wall at end-systole was significantly thinner than at baseline in both infarct groups, however by 10 weeks CDC treatment had restored the wall thickness such that the peri-infarct region in the CDC-treated group was significantly thicker at end systole than that in the control group (control 2.1±0.04 mm, CDC 2.5±0.1 mm, p<0.05 vs control; Figure 3).

### CDC Retention and cardiac morphology

**Figure 4:**
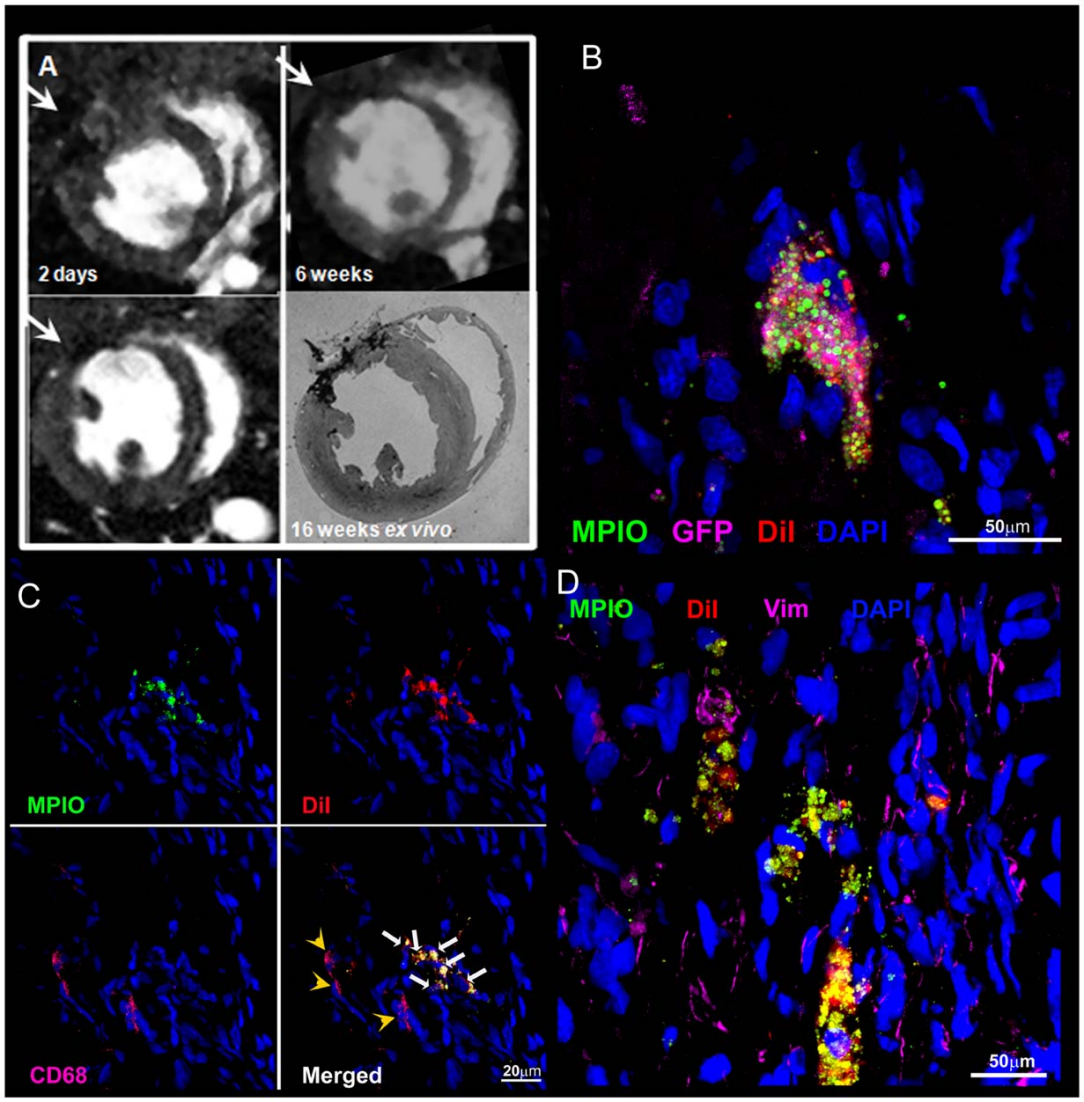
MPIO-labelled CDCs were tracked *in vivo* and identified in tissue sections. A: Regions of hypointensity due to MPIOs (arrows) were detected in CDC-treated hearts using *in vivo* MRI for 16 weeks after administration, and confirmed using *ex vivo* MR microscopy (example images from the same heart at 2 days, 2 weeks and 16 weeks are shown, *in vivo* top, left & right, bottom left, *ex vivo* bottom right). B: In tissue slices, MPIOs (green) were shown to co-localise with expression of GFP (magenta). C: Macrophages were detected in tissue slices, by antibody labelling for CD68 (magenta, yellow arrowheads), but were not labelled with DiI (red) or MPIOs (green, white arrows). D: No vimentin positive/MPIO/DiI positive cells were observed and therefore the cells did not appear to be myofibroblasts.

**Figure 5:**
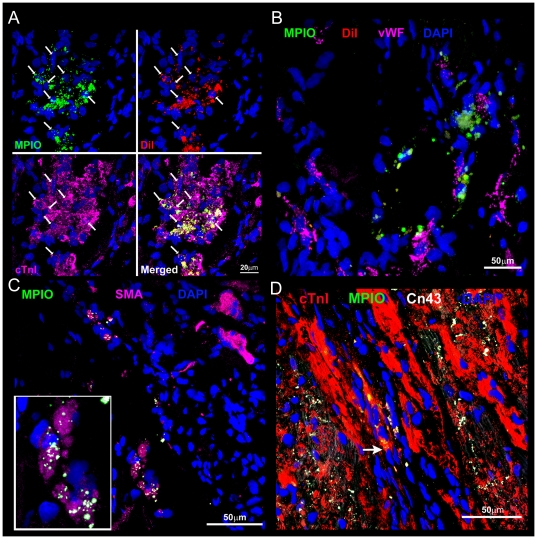
Differentiating CDCs were detected in tissue sections. Regenerating myocardial cells were detected by co-localisation of MPIO (green) and DiI (red) labelling with (A) immuno-staining for cTnI (magenta); (B) vWF-positive capillaries (magenta) and (C) SMA-positive arterioles (magenta, inset is at ×2 zoom), indicating de-novo cardiomyocyte and vessel formation from administered CDCs. (D) MPIO/cTnI positive small immature cells within the border zone expressed a small amount of connexin 43 (arrow), but these cells did not form gap junctions with mature cardiomyocytes.

**Figure 6:**
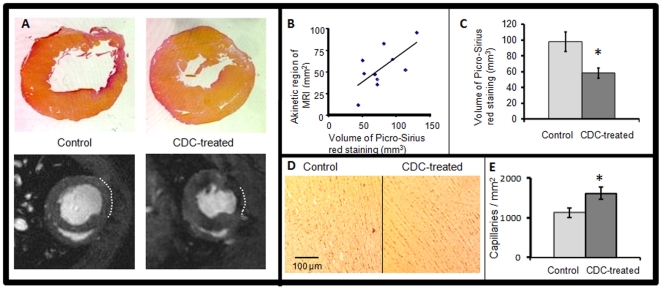
CDC administration reduced infarct size and increased capillary density. Infarct size measurements obtained using picro-Sirius red staining (A: top) and MRI (A: bottom) were found to correlate (B) and showed that CDC-treated hearts were less fibrotic than control hearts (C). Immuno-staining for CD31 showed increased capillary density in the peri-infarct region of CDC-treated hearts compared with controls; representative images are shown in (D) and quantified in (E).

Hypointensities caused by the MPIO particles were detected at all times in the infarct region of CDC-treated hearts using *in vivo* MRI (Figure 4A) and could be measured with high resolution using *ex vivo* microscopy (Figure 4A). The dragon green fluorescence of the MPIOs was clearly detected in tissue slices (Figures 4B–D, 5A–D). Donor cells expressing GFP were detected using antibody labelling, which co-localised with detection of fluorescence from the MPIOs (Figure 4B and Figure S3). Furthermore, although macrophages were detected in tissue slices, by antibody labelling for CD68, they were not labelled with the MPIOs (Figure 4C), showing that MPIO-labelled CDCs survived in the myocardium at 16 weeks and neither they nor the iron particles had been taken up by the infiltrating macrophages. Cells labelled both with MPIOs and with DiI were located in abundance in the fibrotic region of the scar, in the epicardium and, more rarely, in remote myocardium. MPIO-labelled CDCs were not positive for vimentin and therefore the cells did not appear to be myofibroblasts (Figure 4D). Regenerating myocardial cells were detected by co-localisation of immuno-staining for cTnI, vWF, and SMA with MPIO and DiI labelling (Figure 5A–D). 49±3% of MPIO-labelled cells in the infarct/peri-infarct region also expressed cTnI (Figure 5A & D), 12±3% in vWF-positive capillaries (Figure 5B) and 8±2% were detected in SMA-positive arterioles (Figure 5C). The MPIO/cTnI double positive cells remained small in size, indicative of a not fully matured CDC-derived cardiomyocyte. Although these immature cardiomyocytes expressed a small amount of connexin 43, these were not in connection with neighbouring and survived myocytes and therefore no gap junctions were formed (Figure 5D). Infarct size was measured using MR and by histological staining of tissue slices taken to corresponded with the position of the MR imaging slices (Figure 6A). Collagen density, measured using picro-Sirius Red staining, correlated with the size of the akinetic region of the myocardial wall measured in the *in vivo* MR images (Figure 6B). Collagen density was reduced by 40% (p<0.05) in CDC-treated hearts compared with untreated control hearts (Figure 6C). Capillary density, measured using immuno-staining for CD31 (Figure 6D), was increased by 44% (p<0.05) in the peri-infarct region of CDC-treated hearts compared with control hearts (Figure 6E). In summary, CDCs were retained in the infarcted rat heart for at least 16 weeks, increased ejection fraction and capillary density, and ameliorated the increase in end systolic volume and relative infarct size compared with untreated infarcted rat hearts.

## Discussion

Here, we have used MRI to show for the first time that cardiac function was improved and maintained over long-term follow-up in the rat heart following ischaemia/reperfusion and cardiosphere-derived cell therapy.

CDCs are an heterogeneous mixture of cells reported to include c-kit+/CD105+ cardiac progenitor cells, CD90+/CD105+ cardiac mesenchymal cells and CD31+/CD34+ endothelial cells [10]. Andersen *et al* [7] suggested that cells cultured using the explant protocol comprise a mixture of fibroblasts and blood-borne cells, however we found that less than 10% of cells expressed the fibroblast marker DDR2 [18] and less than 1% were CD45+ haematopoietic cells. CDCs from neonatal rat heart were cultured to p2 within one month of biopsy. The percentage of c-kit+ cells at p2 was lower than reported by Smith *et al* [5], with mRNA expression comparable with that shown by Andersen *et al* [7]. Nevertheless, cells expressing the pluripotent markers Oct3/4 and Sox-2 were detected within the CDC population. Li *et al* reported that cardiosphere culture increased expression of Sox-2 and Nanog in human CDCs but that this expression was lower after 3 days of monolayer culture [19]. Furthermore, GATA-4 high c-kit+ cardiac stem progenitor cells (CSCs), which are committed to the cardiomyocyte lineage and show enhanced cardiomyocyte differentiation, have decreased cardiosphere generation capability, compared to GATA-4 low c-kit+ CSCs, which consist of more primitive (Oct-4 and Sox-2 positive) stem cells [20] . The expression of the pluripotent markers, Oct-4 and Sox-2, and the cardiac transcription factors, GATA-4 and Nkx2.5, in CDCs was heterogeneous, but evident in the majority of the cells (Figure 1). Down-regulation of the pluripotent markers is required for the cells to commit to the cardiac lineage, when there is an up-regulation of the cardiac transcription factors, such as Nkx2.5 and GATA-4 [21].

Treatment of CDCs with DMSO for 10 days promoted expression of cTnT, Nkx2.5 and myosin heavy chain, demonstrating their ability to differentiate into more mature myocyte precursors. Finally, the detection of α-smooth muscle actin, von Willibrand factor and α-sarcomeric actin suggested that CDCs contained cells capable of forming all three cardiac lineages. MRI, the gold standard for clinical measurement of cardiac function [22], produces accurate, reproducible measurements of cardiac function in rodents [23] and, in concert with iron oxide labelling of cells, can be used to demonstrate the success of donor cell administration and cell retention [24]. The validity of cell tracking using iron oxide has been questioned, as *in vivo* studies identified iron oxide nanoparticles in macrophages and not in the administered cells [25]. However, larger MPIOs were retained in administrated cells *in vivo* for 3 weeks in CDCs [26] and for 16 weeks in bone marrow cells [24], [27]. Here, MPIOs were found to co-localise with DiI labelling, and expression of GFP confirmed that the majority of the MPIOs were retained within the donor cells 16 weeks after administration. Furthermore, although macrophages were detected in tissue slices, they did not co-localise with the MPIO particles. The MPIOs used here were encapsulated in a polymer which made the surface of the particle free of iron oxide. It may be that these larger, encapsulated particles are retained within the cell more successfully than iron oxide nanoparticles.

The reperfused heart is more appropriate and applicable for pre-clinical studies where cells are administered shortly after infarction. Although human myocardial infarction is followed by reperfusion of the occluded vessel using thrombolytics and/or percutaneous coronary intervention [28], [29], most studies of stem cell therapy in rodents still use total occlusion of the coronary artery [5]. The infarct region following reperfusion differs substantially from that following total occlusion [30]. Reperfusion results in decreased infarct size and reduced remodelling [31], but also in an influx of reactive oxygen species and neutrophils, which cause myocyte necrosis [32]. As a result, the peri-infarct environment differs substantially between the two infarct models. The increased inflammatory reaction immediately following reperfusion may result in clearance of administered donor cells from the peri-infarct region. Therefore, CDCs were also delivered by systemic infusion after two days, when the initial inflammatory influx would have subsided [32].

Here *in vivo* cine MRI provided extensive characterisation of cardiac function in the rat heart immediately after ischaemia/reperfusion and at four time points over the subsequent 16 weeks. Conflicting reports of the efficacy of endogenous cardiac cells cultured from tissue explants may be ascribed to differences in techniques and species. Intracoronary administration of 3×10^6^ porcine CDCs reduced infarct size and improved haemodynamic function in the pig heart 8 weeks after balloon occlusion and reperfusion [6], yet there was no improvement in ejection fraction. After injection of EDCs, expanded without forming cardiospheres, Li *et al* [8] observed no difference in cardiac function, haemodynamics or infarct size at 8 weeks between untreated mouse hearts and those injected with 5×10^5^ cells. *In vivo* bioluminescent imaging showed poor donor cell survival by 8 weeks. In contrast, also in the mouse, Davis *et al* [12] reported a 9% improvement in ejection fraction, measured using MRI, at 3 and 6 weeks after administration of 1×10^6^ EDCs and Chimenti *et al* [11] reported a 16% increase in ejection fraction, measured using 2D echocardiography, at three weeks after administration of 1×10^5^ CDCs. The significant progressive decline in cardiac function we observed in untreated infarcted rat hearts, shown by significantly decreased ejection fraction and increased end systolic volume, was prevented by CDC administration. Accurate measurement of cardiac morphology showed that the systolic wall thickness of the peri-infarct region was comparable in both groups at 2 weeks, but by 10 weeks CDC treatment had restored wall thickness in this region. This may have resulted from the CDC-derived cardiomyocytes, increased capillary density and reduced fibrosis in treated hearts, as also reported by others [11].

The administered CDCs were predominantly localised within the fibrotic regions of the infarct. MPIO- and DiI-labelled cells expressing cTnI, vWF and SMA were detected in the infarct and, more rarely, in the remote myocardium, indicating differentiation of CDCs along the cardiomyocyte lineage and, less frequently, the formation of new blood vessels. However, less than half of the labelled cells within the infarct/peri-infarct region expressed cTnI and these cells were also small in size, suggesting an immature, fetal phenotype. Therefore, the major contribution to the observed long-term improvement in cardiac function most likely resulted from increased paracrine factors that reduced fibrosis and increased capillary density in CDC-treated hearts. Human CDCs secrete vascular endothelial growth factor, hepatocyte growth factor (HGF) and insulin-like growth factor 1 (IGF-1) leading to increased capillary density in the mouse heart 3 weeks after CDC administration [11]. Moreover, administration of HGF and IGF-1 to the infarcted pig heart resulted in activation and differentiation of endogenous c-kit^+^ cardiac stem cells as well as improved cardiomyocyte survival, and reduced fibrosis and cardiomyocyte hypertrophy [33]. Further investigation is warranted to identify factors and conditions that promote the maturation of the CDC-derived cardiomyocytes to a mature and functional phenotype within the infarcted region.

Despite producing a CDC population containing about 30% more CD90+ cardiac mesenchymal cells and 12% fewer c-kit+ cardiac progenitor cells than others [5], we found that a single injection of 2×10^6^ CDCs 10 minutes post ischaemia/reperfusion MI and a further bolus of 4×10^6^ CDCs administered systemically 2 days later, homed to the infarcted myocardium and were retained at 16 weeks follow-up. A significant proportion of CDCs differentiated towards the cardiomyocyte lineage. We also found lower fibrosis and increased capillary density at 16 weeks, suggesting the release of paracrine factors by the transplanted CDCs. Importantly, these changes resulted in improved cardiac function at 4 months, which was not evident in the control group that did not receive CDC treatment. Thus, cardiosphere-derived cells may be expanded from biopsy samples to provide a cell population capable of preserving long-term function in the infarcted heart.

## Supporting Information

File S1 **Materials and Methods.**
(PDF)

Figure S1 **Confluent CDCs (top) and cardiospheres (bottom) contained high levels of CD90+ cardiac mesenchymal cells.** Cardiospheres also contained low levels of spontaneously differentiating cells, detected by staining for cTnI.
(PDF)

Figure S2 **CDCs cultured to confluency (middle panels) showed spontaneous differentiation with expression of cTroponin T and α-sarcomeric actin, which were not seen in non-confuent CDCs (top panels).** Culture in differentiation medium containing DMSO (bottom panels) resulted in cells adopting an elongated morphology more indicative of a cardiomyocyte and beginning to show striations.
(PDF)

Figure S3 **Detection of GFP-expressing cells labelled with DiI and MPIOs confirmed that administered cells were retained in the hearts after 16 weeks; the white arrows identify double stained GFP+ MPIO+ CDCs, whilst the yellow arrowheads identify GFP+ CDCs alone.**
(PDF)

Table S1 **Cardiac function and morphology measured over 16 weeks using MRI.**
(PDF)
