# Supplementary material for: Cardiosphere-Derived Cells Improve Function in the Infarcted Rat Heart for at Least 16 Weeks – an MRI Study
Source: PLoS One. 2011 Oct 17;6(10):e25669. doi: 10.1371/journal.pone.0025669 (PMC3197153; doi:10.1371/journal.pone.0025669)
Supplement: File S1 — Materials and Methods. (PDF) [file pone.0025669.s001.pdf]

**Cardiosphere-derived cells improve function in the infarcted rat heart for at least 16 weeks – an MRI study**

CA Carr[1], DJ Stuckey[1,2], JJ Tan[1,3], SC Tan[1], RSM Gomes[1], P Camelliti[1,2], E Messina[3], A Giacomello[3], GM Ellison[4], K Clarke[1]

**Supplementary Information File S1 – Materials and Methods.**

**RNA extraction and DNase treatment**

Total RNA was extracted from cultured CDCs using Trizol reagent (Sigma) according to the manufacturer's instructions. DNase treatment was performed using Turbo DNA-free (Ambion) to degrade any DNA present. The concentration and purity of RNA was determined by measuring the absorbance at 260 nm (A260) and 280 nm (A280) using a Nanodrop ND-1000 Spectrophotometer (Nanodrop Technologies Inc., USA). A ratio of A260/A280 ≈ 2.0 is generally accepted as pure RNA.

**cDNA synthesis**

DNase treated RNA was reverse transcribed using AB high capacity transcriptase kit (Applied Biosystem). In brief, every 1µg RNA sample was reverse transcribed using 1 µl reverse transcriptase, 2 µl random primer, 0.8 µl dNTPs (10mM each), 2 µl buffer and topped up by RNase free water to a total volume of 20 µl. The reaction mixture was subjected to incubation for 10 minutes at 25°C, 120 minutes at 37°C and 5 seconds at 85°C.

**Real-time PCR**

The real-time PCR mastermix was prepared by adding 10 µl AB Sybrgreen PCR mastermix (AB International, CA), 1 µl reverse primer, 1 µl forward primer, 1 µl cDNA and 7 µl distilled water. The PCR program was set up with an initial heat activation step at 95°C for 10 minutes. Thermocycling (40 cycles) was performed with a denaturation step at 95°C for 15 seconds, an annealing step at 60°C for 30 seconds

and an extension step at 72°C for 30 seconds. Fluorescence was measured at the end of each extension step.

After amplification, a melting curve was acquired by heating the product at 4°C/second to 95°C. Fluorescence was measured through the slow heating phase. Melting curves were used to determine the specificity of PCR products.

**Primer Sequences**

| **Primer** | **Gene name** | **Forward primer 5' to 3'** | **Reverse primer 3' to 5'** | **Gene accession number** |
|---|---|---|---|---|
| c-Kit | Stem cell factor cytokine receptor | AATCCGACAAC CAAAGCAAC | TGACATCAGAG TTGGACACCA | ENSRN-OG00000002227 |
| GATA 4 | GATA binding protein 4 | CAGTCCTGCAC AGCCTACCT | CCGCAGTTGAC ACACTCTCT | NM_144730 |
| CD90 | Thy-1 cell surface antigen | CAGAATCCCAC AAGCTCCAA | GCCAGGAAGTG TTTTGAACC | NM_012673 |
| CD105 | Endoglin | GGTACAGTGCA TCGACATGG | GCTGGCCTAGC TCTATGGTG | NM_001010968 |
| Nkx2.5 | Homeobox protein NKX2-5 | CATTTTATCCG CGAGCCTAC | GTCTGTCTCGG CTTTGTCCA | ENSRNOG000000 20747 |
| Tnnt 2 | Troponin T type 2 (cardiac) | CGTATTCGCAA TGAACGAGA | CTGTTCTCCTC CTCCTCACG | NM_012676 |
| MyHC | Myosin heavy chain | TATGAGACGGA CGCCATACA | CTCCAGAGAGG AGCACTTGG | NM_017239.2 |
| GAPDH | glyceraldehyde-3-phosphate dehydrogenase | GGGTGTGAACC ACGAGAAAT | ACTGTGGTCAT GAGCCCTTC | NM_017008 |

***In vivo* cine MRI**

1.5 mm true short axis ECG-gated cine images were acquired with the following parameters: field of view 51.2 mm$^2$, matrix size 256 x 256 zero filled to 512 x 512 giving a voxel size of 100 x 100 x 1500 µm, echo time/repetition time (TE/TR) 1.43/4.6 ms, 17.5° pulse, 25-35 frames per cardiac cycle

Stroke volume was calculated as end diastolic volume minus end systolic volume. The ejection fraction was calculated as the stroke volume divided by the end diastolic volume. The relative infarct size was calculated from the average of the

endocardial and epicardial circumferential lengths of the thinned, akinetic region of all slices, measured at diastole, and expressed as a percentage of the total myocardial surface [1]. Wall thickness of the peri-infarct (myocardium adjacent to infarct where contraction was observed) and posterior regions of the myocardium was measured at end systole in a mid-papillary slice. Myocardial mass was calculated from an average of the myocardial volumes at end diastole and end systole, multiplied by the density of myocardial tissue (1.05 mg/ml) [2].

**High resolution 3D MR microscopy**

MR microscopy was performed using a fast gradient echo sequence with the following parameters: TE/TR = 1.8/30 ms, flip angle 90º, field of view: 32 x 32 x 32 mm, matrix size 512 x 512 x 512, voxel size $62.5 \times 62.5 \times 62.5$ µm.

**Antibodies for immunohistochemistry and flow cytometry**

| Antigen | Antibody | Supplier | Catalogue number | Code | Dilution |
|---|---|---|---|---|---|
| Alpha-sarcomeric actin | Mouse monoclonal | Sigma | A2172 | 5C5 | 1:200 |
| CD31 | Mouse monoclonal | Serotec | MCA1334G | TLD-2A12 | 1:100 |
| CD68 | Mouse monoclonal | Abcam | AB31630 | ED1 | 1:200 |
| CD90 | Mouse monoclonal | BD Pharmingen | 554892 | HIS51 | 1:250 |
| CD117 (c-kit) | Rabbit polyclonal | Santa Cruz | SC-5535 | H300 | 1:50 |
| Connexin 43 | Mouse monoclonal | Sigma | C8093 | CXN-6 | 1:200 |
| DDR2 | Goat polyclonal | Santa Cruz | SC-7555 | N20 | 1:20 |
| GFP | Goat polyclonal | Rockland | 600-101-215 | | 1:50 |
| GATA-4 | Rabbit polyclonal | Santa Cruz | SC-9053 | H-112 | 1:50 |
| Klf-4 | Rabbit polyclonal | Santa Cruz | SC20691 | H-180 | 1:50 |
| Nanog | Rabbit polyclonal | Abcam | Ab21603 | | 1:50 |
| Nkx2.5 | Goat polyclonal | R&D | AF2444 | | 1:50 |
| Oct 3/4 | Rabbit polyclonal | Santa Cruz | SC-9081 | H134 | 1:50 |
| Smooth muscle actin | Mouse monoclonal | Sigma | A2547 | 1A4 | 1:500 |

| | | | | | |
|---|---|---|---|---|---|
| Sox 2 | Goat polyclonal | Santa Cruz | SC-17320 | Y17 | 1:50 |
| Cardiac troponin I | Rabbit polyclonal | Santa Cruz | SC-15368 | H170 | 1:500 |
| Cardiac troponin T | Mouse monoclonal | Abcam | Ab10214 | 1F11 | 1:200 |
| Vimentin | Mouse monoclonal | Sigma | V2258 | LN-6 | 1:200 |
| von Willebrand Factor | Rabbit polyclonal | Chemicon | AB-7356 | | 1:200 |
| MitoTracker Red CMXRos | | Jackson Immuno Research | M7512 | | 50 nm |

1. Nahrendorf M, Wiesmann F, Hiller KH, Hu K, Waller C, et al. (2001) Serial cine-magnetic resonance imaging of left ventricular remodeling after myocardial infarction in rats. J Magn Reson Imaging 14: 547-555.
2. Tyler DJ, Lygate CA, Schneider JE, Cassidy PJ, Neubauer S, et al. (2006) CINE-MR imaging of the normal and infarcted rat heart using an 11.7 T vertical bore MR system. J Cardiovasc Magn Reson 8: 327-333.
