# Supplementary material for: Cardiosphere-Derived Cells Improve Function in the Infarcted Rat Heart for at Least 16 Weeks – an MRI Study
Source: PLoS One. 2011 Oct 17;6(10):e25669. doi: 10.1371/journal.pone.0025669 (PMC3197153; doi:10.1371/journal.pone.0025669)
Supplement: Figure S1 — Confluent CDCs (top) and cardiospheres (bottom) contained high levels of CD90+ cardiac mesenchymal cells. Cardiospheres also contained low levels of spontaneously differentiating cells, detected by staining for cTnI. (PDF) [file pone.0025669.s002.pdf]

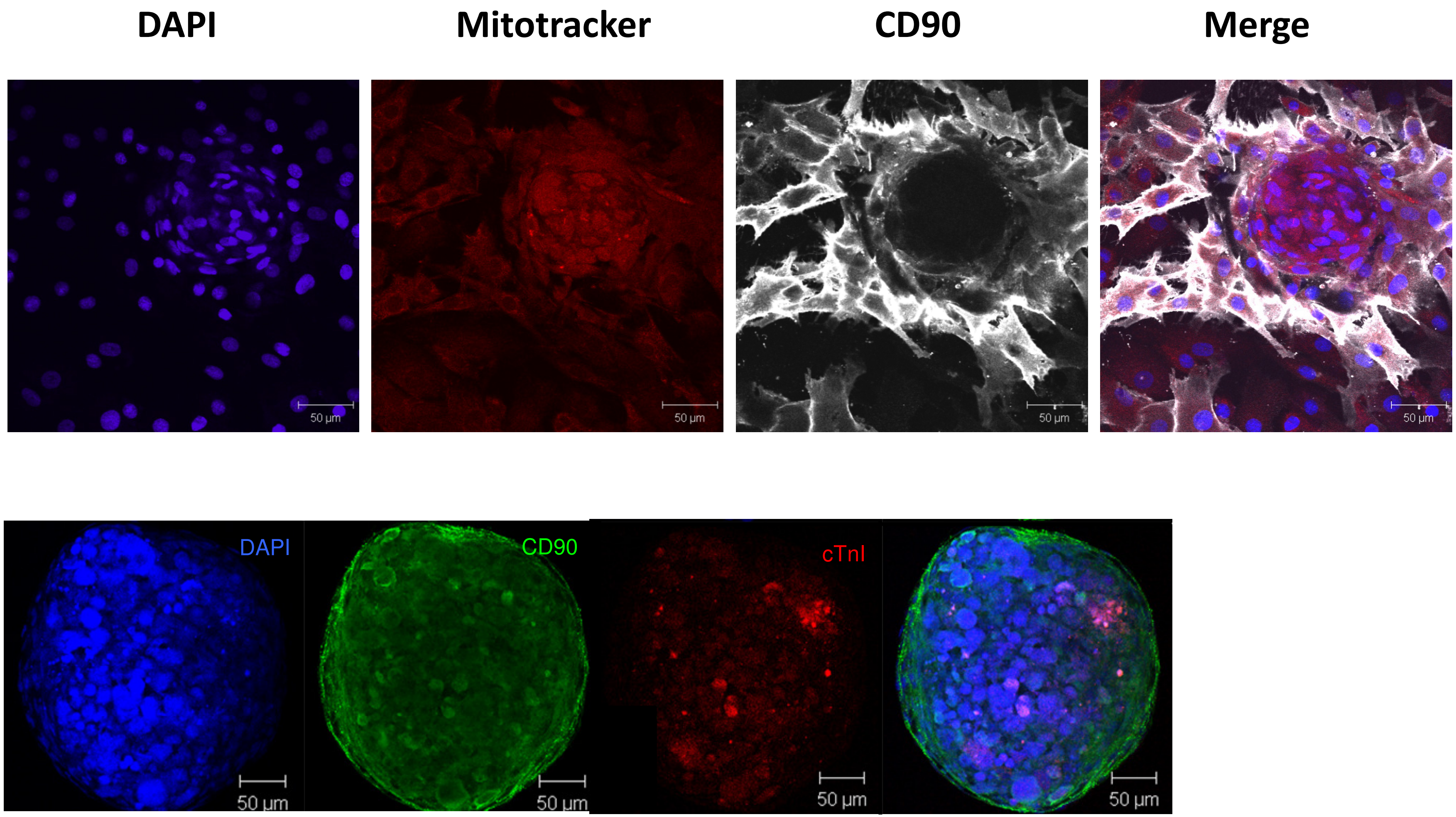

Supplementary figure S1: confluent CDCs (top) and cardiospheres (bottom) contained high levels of CD90+ cardiac mesenchymal cells. Cardiospheres also contained low levels of spontaneously differentiating cells, detected by staining for cTnI.
