# Supplementary material for: Cardiosphere-Derived Cells Improve Function in the Infarcted Rat Heart for at Least 16 Weeks – an MRI Study
Source: PLoS One. 2011 Oct 17;6(10):e25669. doi: 10.1371/journal.pone.0025669 (PMC3197153; doi:10.1371/journal.pone.0025669)
Supplement: Figure S2 — CDCs cultured to confluency (middle panels) showed spontaneous differentiation with expression of cTroponin T and α-sarcomeric actin, which were not seen in non-confuent CDCs (top panels). Culture in differentiation medium containing DMSO (bottom panels) resulted in cells adopting an elongated morphology more indicative of a cardiomyocyte and beginning to show striations. (PDF) [file pone.0025669.s003.pdf]

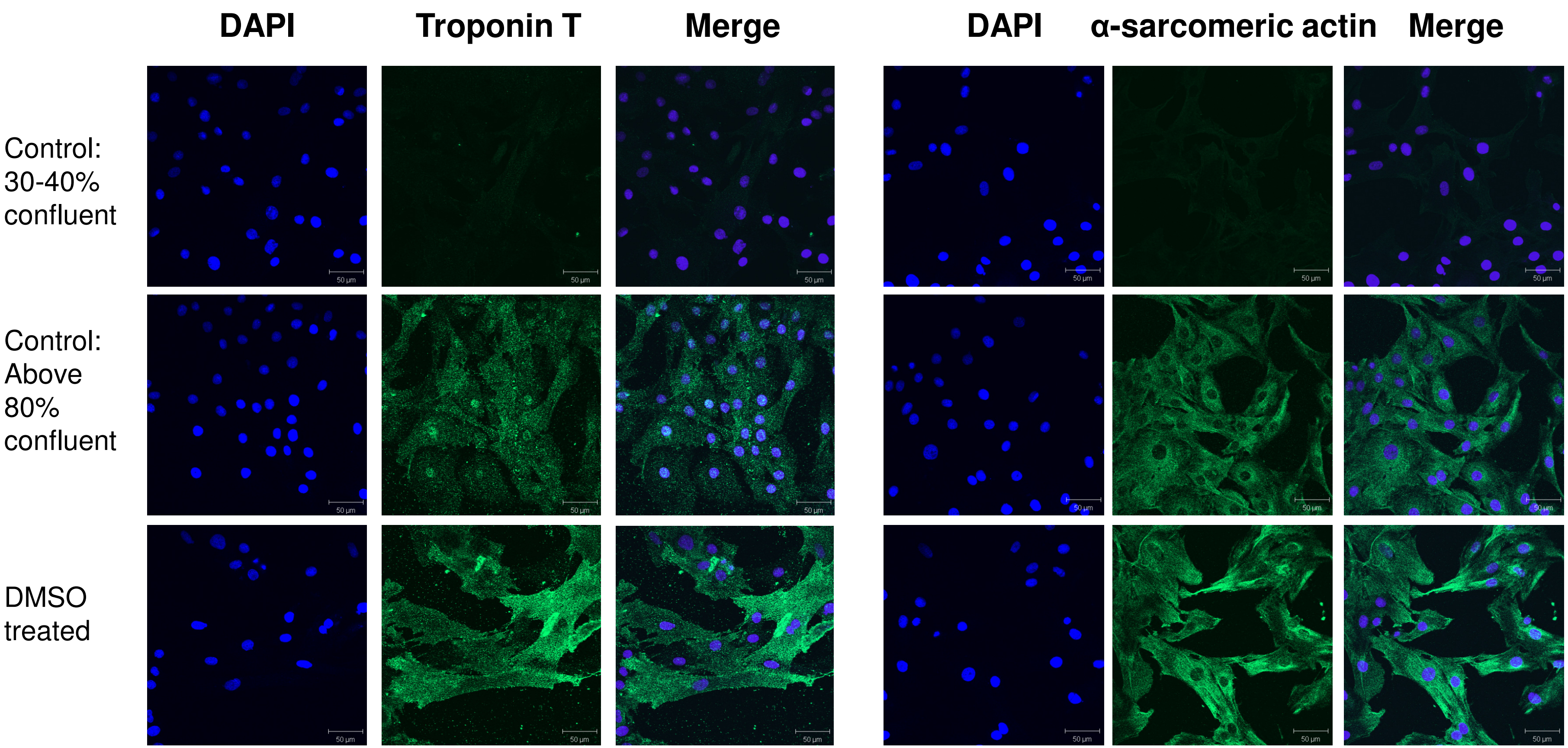

Supplementary figure S2: CDCs cultured to confluency (middle panels) showed spontaneous differentiation with expression of cTroponin T and α-sarcomeric actin, which were not seen in non-confuent CDCs (top panels). Culture in differentiation medium containing DMSO (bottom panels) resulted in cells adopting an elongated morphology more indicative of a cardiomyocyte and beginning to show striations.
