# Supplementary material for: Cardiosphere-Derived Cells Improve Function in the Infarcted Rat Heart for at Least 16 Weeks – an MRI Study
Source: PLoS One. 2011 Oct 17;6(10):e25669. doi: 10.1371/journal.pone.0025669 (PMC3197153; doi:10.1371/journal.pone.0025669)
Supplement: Figure S3 — Detection of GFP-expressing cells labelled with DiI and MPIOs confirmed that administered cells were retained in the hearts after 16 weeks; the white arrows identify double stained GFP+ MPIO+ CDCs, whilst the yellow arrowheads identify GFP+ CDCs alone. (PDF) [file pone.0025669.s004.pdf]

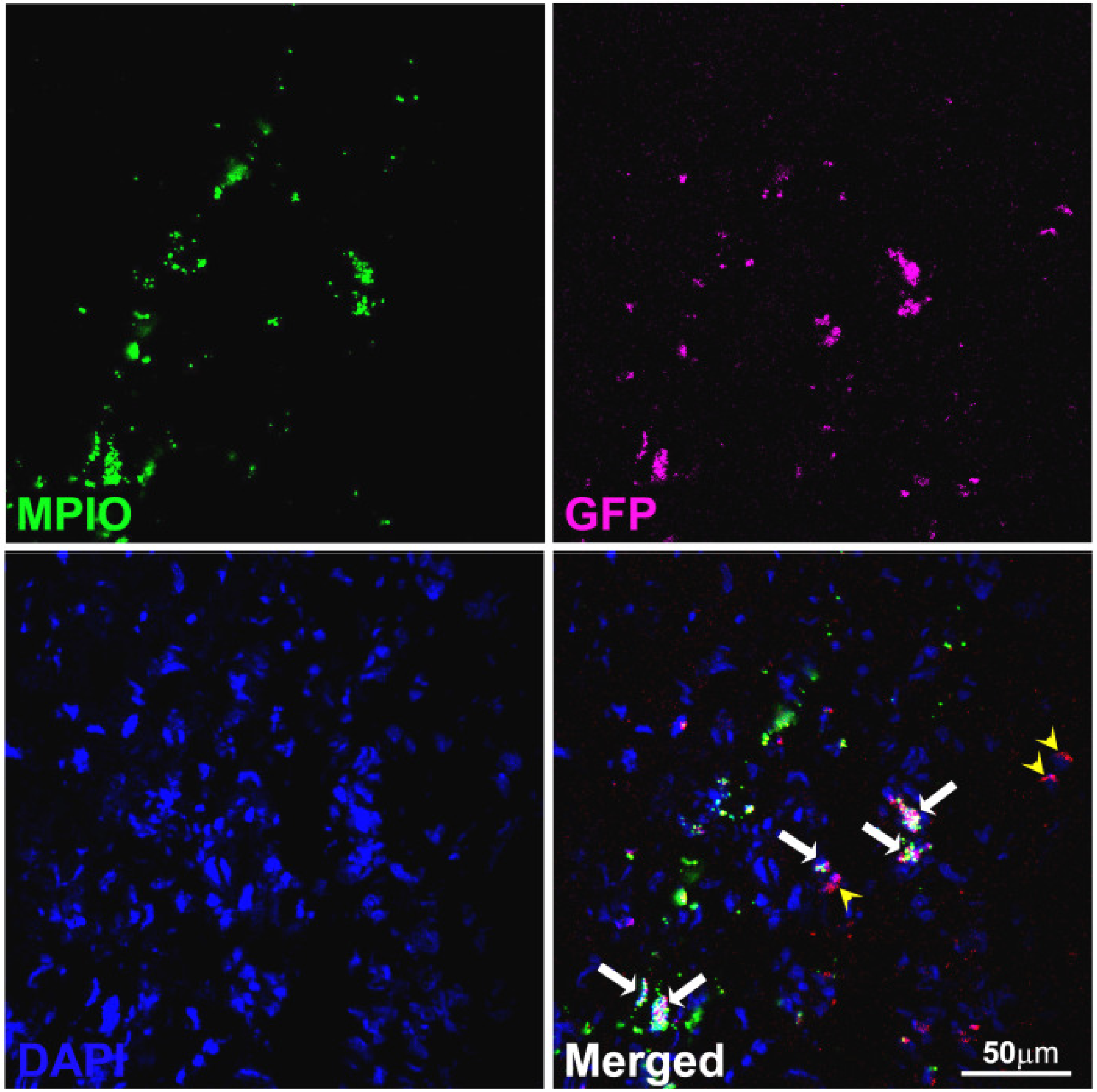

Supplementary figure S3: Detection of GFP-expressing cells labelled with DiI and MPIOs confirmed that administered cells were retained in the hearts after 16 weeks; the white arrows identify double stained GFP+ MPIO+ CDCs, whilst the yellow arrowheads identify GFP+ CDCs alone
