# Supplementary material for: Cardiosphere-Derived Cells Improve Function in the Infarcted Rat Heart for at Least 16 Weeks – an MRI Study
Source: PLoS One. 2011 Oct 17;6(10):e25669. doi: 10.1371/journal.pone.0025669 (PMC3197153; doi:10.1371/journal.pone.0025669)
Supplement: Table S1 — Cardiac function and morphology measured over 16 weeks using MRI. (PDF) [file pone.0025669.s005.pdf]

Cardiosphere-derived cells improve function in the infarcted rat heart for at least 16 weeks – an MRI study

CA Carr[1], DJ Stuckey[1,2], JJ Tan[1,3], SC Tan[1], RSM Gomes[1], P Camelliti[1,2], E Messina[3], A Giacomello[3], GM Ellison[4], K Clarke[1]

Supplementary Table S1: Cardiac function and morphology measured over 16 weeks using MRI

* p < 0.05 vs sham ; # p < 0.05 vs control; † p < 0.05 vs 2 days (shown only for infarct area for clarity)

	2 days			2 weeks			6 weeks			10 weeks			16 weeks		
	Sham	Control	CDC	Sham	Control	CDC	Sham	Control	CDC	Sham	Control	CDC	Sham	Control	CDC
**Ejection Fraction (%)**	72 ± 4	60 ± 5	62 ± 3	68 ± 1	52 ± 2 *	55 ± 2 *	68 ± 3	47 ± 2 *	56 ± 3 *#	64 ± 1	49 ± 3 *	57 ± 3 #	67 ± 2	47 ± 3 *	57 ± 3 #
**End Systolic Volume (mm^3)**	73 ± 17	122 ± 20	112 ± 11	76 ± 9	247 ± 27 *	177 ± 13 *#	113 ± 11	301 ± 42 *	211 ± 24	142 ± 18	322 ± 59	200 ± 23	125 ± 4	360 ± 65 *	224 ± 33
**End Diastolic Volume (mm^3)**	269 ± 39	298 ± 18	288 ± 16	238 ±20	516 ± 64 *	392 ± 15	352 ± 10	579 ± 92	469 ± 29	392 ± 39	621 ± 106	461 ± 29	383 ± 22	671 ± 103	512 ± 40
**Stroke Volume (mm^3)**	196 ± 23	176 ± 11	176 ± 8	162 ± 11	269 ± 39	216 ±6	239 ± 10	278 ± 52	258 ± 8	250 ± 21	299 ± 51	260 ± 13	258 ± 22	311 ± 41	288 ± 11
**Heart rate (bpm)**	361 ± 13	379 ± 15	354 ± 17	368 ± 36	341 ± 13	350 ± 16	403 ± 3	314 ± 15	342 ± 16	350 ± 18	323 ± 13	358 ± 14	377 ± 19	330 ± 9	340 ± 12
**Cardiac Output (ml/min)**	67 ± 2	66 ± 4	62 ± 4	59 ± 2	90 ± 9 *	76 ± 5	83 ± 9	86 ± 9	89 ± 6	87 ± 7	94 ± 13	93 ± 4	98 ± 13	102 ± 12	98 ± 3
**Relative infarct size (%)**	-	9 ± 1	9 ± 2	-	12 ± 2	12 ± 1	-	13 ± 2	11 ± 1	-	15 ± 2 †	12 ± 2	-	16 ± 2 †	10 ± 1 #
**Infarct area (mm^2)**		24 ± 4	24 ± 4		43 ± 11	36 ± 4		47 ± 9	37 ± 5 †		56 ± 4 †	38 ± 4 †		61± 9 †	36 ± 7 #
**End systolic posterior wall thickness (mm)**	2.5 ± 0.1	2.5 ± 0.1	2.6 ± 0.1	2.6 ± 0.1	2.5 ± 0.2	2.4 ± 0.1	2.6 ± 0.2	2.3 ± 0.1	2.3 ± 0.2	2.3 ± 0.2	2.4 ± 0.2	2.4 ± 0.2	2.4 ± 0.1	2.6 ± 0.2	2.4 ± 0.2
**End systolic peri-infarct wall thickness (mm)**	-	2.7 ± 0.2	2.8 ± 0.1	-	1.9 ± 0.2 †	2.2 ± 0.1 †	-	2.0 ± 0.2 †	2.2 ± 0.1 †	-	2.1 ± 0.2 †	2.5 ± 0.2	-	2.1 ± 0.0 †	2.5 ± 0.1 #
**Myocardial mass (mg)**	423 ± 35	553 ± 20	588 ± 41 *	387 ± 14	597 ± 19 *	566 ± 23 *	515 ± 47	648 ± 35	638 ± 29	491 ± 19	686 ± 51 *	640 ± 23	519 ± 30	748 ± 36 *	702 ± 35 *
**Body weight (g)**	227 ± 5	207 ± 11	232 ± 5	234 ± 9	201 ± 13	247 ± 6	259 ± 6	235 ± 8	263 ± 7	275 ± 7	242 ± 7	261 ± 8	309 ± 11	256 ± 6	273 ± 5
**Heart weight/body weight (x 10^3)**	1.9 ± 0.2	3.1 ± 0.3 *	2.5 ± 0.2	1.7 ± 0.2	2.6 ± 0.1 *	2.3 ± 0.1	2.0 ± 0.2	2.8 ± 0.2	2.4 ± 0.1	1.8 ± 0.1	2.7 ± 0.2 *	2.5 ± 0.1 *	1.7 ± 0.0	2.8 ± 0.2 *	2.6 ± 0.1 *
